# The lunar-tide cycle viewed by crustacean and mollusc gatherers in the State of Paraíba, Northeast Brazil and their influence in collection attitudes

**DOI:** 10.1186/1746-4269-2-1

**Published:** 2006-01-04

**Authors:** Alberto K Nishida, Nivaldo Nordi, Rômulo RN Alves

**Affiliations:** 1 Departamento de Sistemática e Ecologia, Universidade Federalda Paraíba, 58051-900 João Pessoa, PB, Brasil; 2 Departamento de Hidrobiologia, LEHE, Universidade Federal de São Carlos, Rod. Washington Luiz, Km 235, 13565-905 São Carlos, SP, Brasil; 3 Departamento de Biologia, Universidade Estadual da Paraíba and Programa de Pós-Graduação em Ciências Biológicas (Zoologia), Departamento de Sistemática e Ecologia, Universidade Federal da Paraíba, 58059-970 João Pessoa, PB, Brazil

## Abstract

Traditional human communities have a wide knowledge of their environment. Collection of animals in estuarine and coastal areas are directly influenced by tidal cycles. The aim of this study is to evaluate the understanding of the tides associated with the lunar cycle held by people who gather crustaceans and molluscs in the State of Paraiba. The empirical knowledge of 20 crab gatherers and 30 mollusc gatherers was recorded through open interviews and structured questionnaires. The results showed that the gatherers have an accurate comprehension of tidal phenomenon based on their exploitation of natural resources, which perpetuates through generations.

## Introduction

The diversity of coastal ecosystems and their abundance of natural resources have attracted human communities since ancient times. In Brazil, artisanal fishing is fundamentally relevant because this activity produces food, for commercial purposes and a means of subsistence [1,2]. The bulk of Brazilian fishing is based on mangrove ecosystems whose species spend most of their life cycle in those environments [3].

Human communities relying directly on their natural resources for subsistence, usually have a detailed knowledge of their environments [4-6]. The economic, social, and cultural activities of those people depend upon the local biota and the implicit environmental cycles [7]. Artisanal fishing requires that the participants have ethnoecological knowledge allowing them to use the fishery resource with the sustainability of the *praxis *guaranteed [8,9]. This information is a valuable heritage to be systematized and used to develop measures for a sustainable use of the natural resources [10].

The daily observations of fishermen on the resources and fishing environments result in an accumulation of knowledge that will support ecological studies [11,12]. There are several terms used to describe this type of knowledge, such as 'local', 'traditional' or 'indigenous', depending on the characteristics of the holders of that knowledge [4]. Difficulties regarding this terminology have been addressed in the human ecology and ethnoscience literature (for example Berkes [4]). Ruddle [13] argued that the term 'local' is less problematic, and thus a more practical description or identifier of the relevant people and their knowledge. Ultimately there may be no single terminology that is applicable to all circumstances. According to Faulkner and Silvano [14], the term traditional knowledge is consistent in Brazilian artisanal or local context. For the purposes of this paper the definition offered by Berkes [15] is appropriate, with an additional note emphasizing the fishery-related context of the discussion. Thus traditional fishers' knowledge refers to the fishery-related component of the 'cumulative body of knowledge, practice, and belief, evolving by adaptive processes and handed down through generations by cultural transmission, about the relationship of living beings (including humans) with one another and with their environment' [15].

In recent years, researchers have emphasized the importance of the knowledge produced and orally transmitted by traditional fishermen and the potential role of traditional fishing and related environmental knowledge can play for the development and implementation of fisheries management in the modern world [1,16,17]. Ecologists and environmental managers have generally disregarded the possibilities of learning from the traditional human communities [18]. However, a recent acknowledgment of their relevance has led to an intensification of studies on popular wisdom [12].

In Brazil, research involving ecological knowledge of artisanal fishers has been done mostly over the past ten years, focusing on marine and freshwater fish. Environmental and socio-cultural factors threaten the maintenance of this alternate information base, and serve to highlight the need for increased research efforts to record this knowledge and realize its potential contribution to fisheries management [14].

Tidal variations influence the life cycle of animals inhabiting the coastal zones, acting directly on the general pattern of activities, as for example the land crab (*Ucides cordatus*) studied by Alves and Nishida [19]. The tides are significant abiotic factors determining the pattern of distribution of benthic organisms in estuarine areas. They act on animals' behaviour and also exert decisive roles in fishing strategies adopted by the riparian human populations [10,20,21].

As stated by Maneschy [22] all estuarine and coastal zones are influenced by the tide cycles. Kulesza [23] states that the tides are part of the existence of people who live close to the sea, emphasizing that human populations use their practical wisdom for predicting events related to fishing and navigation. Some fishermen, as pointed out by Ruddle [13], produce calendars and mental maps that enable them to localize fishes according to the phases of the moon.

Crustaceans and molluscs are important to the income of a significant part of the people living close to mangrove ecosystems in the state of Paraíba [19,24,25]. Like other forms of artisanal fishery, crab and mollusc gathering is influenced by lunar-tide cycles.

Despite the relationships between fishing and collection of animals in Brazilian estuarine ecosystems and the tide movements associated, the literature on this subject is scarce. Few studies have been performed on this aspect [10,19,21,22,25-29]. In this work we investigate the knowledge of crustacean and mollusc gatherers with regard to tidal variations and the influence of tides on collection activities.

## Methods

### Study areas

**Figure 1:**
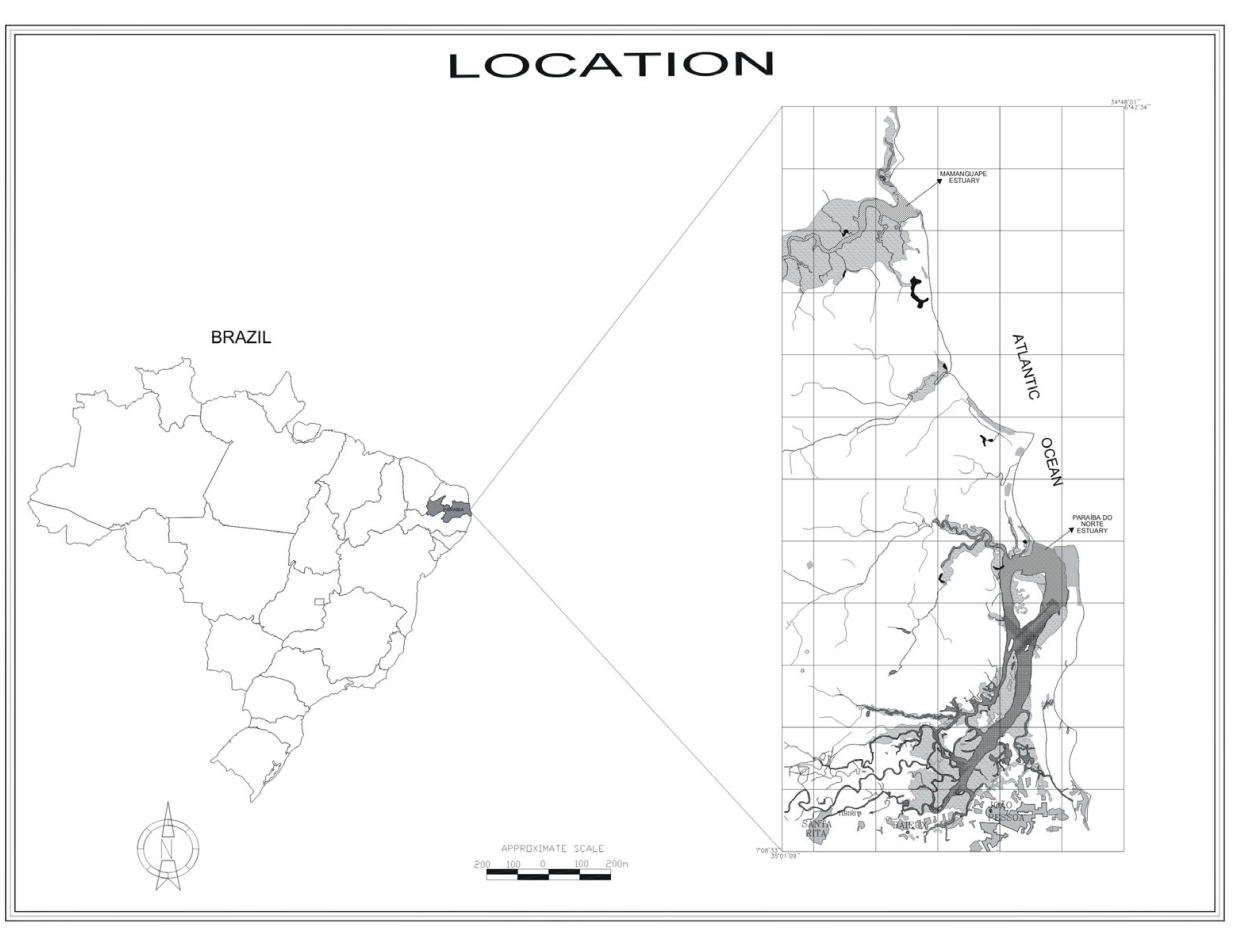
Map of Brazil showing in the inset the coast of Paraíba state with the study areas.

We investigated the communities of artisanal gatherers inhabiting the two largest estuaries of the state of Paraiba, Northeast Brazil: the estuaries of the most important rivers, Mamanguape and Paraíba do Norte (Fig. 1). Artisanal gatherers are commercial and subsistence gatherers alike. The study areas are described as follows.

#### The estuary of the Mamanguape river

**Figure 2:**
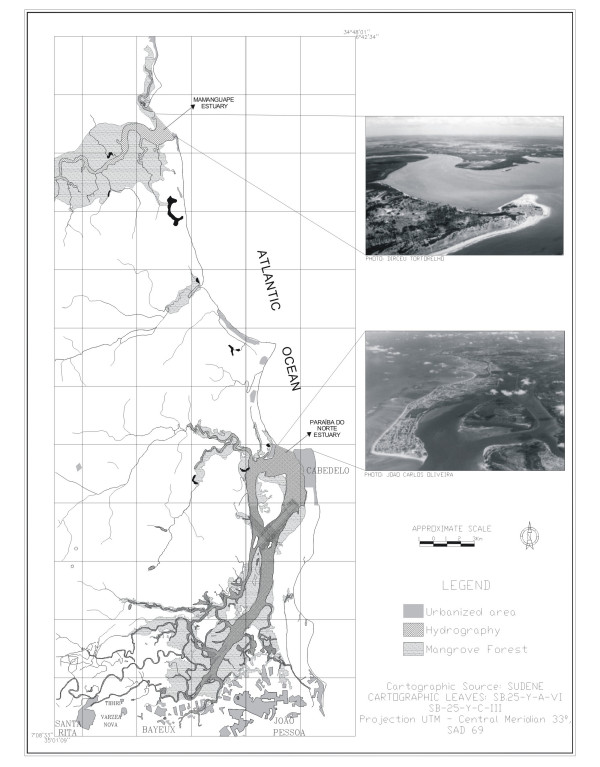
The estuaries of rivers Mamanguape and Paraíba do Norte. The photos show aerial views of the estuaries.

This estuary is located between lat 06°48' and lat 06°51' S and between long 35°07' and 34°54' W. From east to west its inlet is *ca*. 24 km long and it has a maximum width of 2.5 km close to its mouth (Fig. 2). All the area under the influence of the Mamanguape River is within the permanently protected area ('APA' in Portuguese) of the IBAMA (the Brazilian Institute for the Environment and Natural Resources), within which lie islands and islets (the latter are formed by sandy-clay-loam), as well as mangroves.

The estuary of the Mamanguape River and the mangrove forest associated, are important economical and social means of subsistence to nearby human communities. Paludo and Klonowski [30] observed a miscegenation of white, native and black peoples in that region, living in close connection with Nature. They depend upon the mangrove ecosystem for their subsistence and maintenance of their cultural patterns. Vidal [31] reported agricultural activities and animal husbandry in the APA (the permanent protect area). Tourism is also an activity that has been recently started at areas closer to the mouth of that river.

The mangrove ecosystem of the Mamanguape River spreads over *ca*. 6.000 ha composed mainly of *Rhizophora mangle *L., *Avicennia germinans *(L.) Stearn, *Avicennia schaueriana *Stapf & Leechman, *Laguncularia racemosa *Gaertn. f., and *Conocarpus erectus *L[30]. Despite being considered as one of the most well-preserved mangrove areas in Paraíba [32], this estuary currently presents some sites that appear to be affected by expansions of sugar cane plantations. Watanabe *et al*. [33] reported evidences of water contamination in one of the tributaries due to agrochemicals used in sugar cane plantations. Local fishermen state that the agrochemicals have decreased fish production along the Mamanguape River. The islands and islets are also being modified by the increasing siltation of the river bed.

#### The estuary of the Paraíba do Norte river

The basin of the most important river of Paraíba extends for *ca*. 380 km, crossing 37 municipalities. It is divided in Alto (High), Médio (Median), and Baixo (Low) Paraíba [34]. The Baixo Paraíba is located between lat 60°57' and 70°08' S and between long 34°50' and 34°55' W. It drains areas in the municipalities of João Pessoa (the capital of the state of Paraíba), Bayeux, Santa Rita, and Cabedelo (Fig. 2).

In the last ten years the exploitation of resources from the natural estuary-mangrove system has been intensified mainly due to the increasing population in the periphery of João Pessoa. A large part of the population living on the left bank of the river exploits those resources for food and income. On the right bank, where urbanization has been intensified, there is a reduced number of people which depends on the estuarine resources [35].

When compared to the basin of the Mamanguape River, the basin of the Paraíba do Norte River appears more impacted, certainly because it is closer to big cities [25].

### Procedures

The field research was carried out from January 1997 to December 2001, by visiting the human communities described above. Before starting the field work, it was held a meeting with crab and mollusc gatherers to inform them about our goals and methods of study, and to propose their participation in our investigations.

The empirical knowledge of crustacean and mollusc gatherers regarding tides and their relationship to the phases of the moon were obtained by applying semi-structured questionnaires [36,37], complimented by semi-directive interviews and informal conversations [38]. With the informer's permission the interview was recorded then transcribed and submitted to the informer for confirmation.

Twenty experienced crab gatherers and 30 experienced mollusc gatherers were interviewed, making up a stratified sampling. Only crab and mollusc gatherers living exclusively from such activity for at least 20 years were interviewed. Some of gatherers selected were followed during their collection activities. It was then observed that they organize their activities according to tidal variations. Species of crabs and molluscs exploited by gatherers were collected and identified, and subsequently deposited in the Laboratory of Invertebrates of the Systematic and Ecology Department of the Federal University of Paraiba.

The procedures adopted in order to respect indigenous intellectual property rights, led us to follow an informal protocol: before the survey, we briefly explained the research nature and objectives to each gatherer interviewed. The incipient community organization of gatherers [24,25], as well as illiteracy found among many of them, precluded a formal approach using interview consent forms.

All the information obtained from them was verified by directly observing the lunar cycle and its effect on the mangrove ecosystem, and also by comparing the results with the published literature [26,39,40].

We used the native visualization, avoiding this way, the introduction of comments or terms used by the researcher (or interviewer) for not influencing the answer given by the informants [41,42].

## Results and discussion

For many human communities living close to estuaries in the Paraíba, molluscs and crustaceans gathering is the most important means of subsistence, despite their supplemental income from agriculture activity, in small scale, and extraction of other natural resources. Gatherers are economically marginal groups, extremely poor and ignored by other artisanal fishermen. The animals they capture are important for the regional cuisine, and they are usually commercialized at urban centres by resellers.

**Figure 3:**
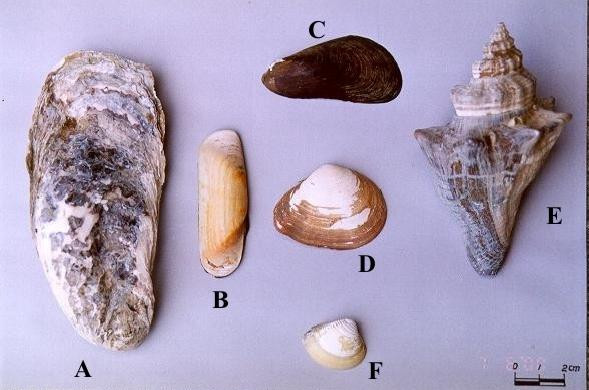
Molluscs exploited commercially in the littoral of the state of Paraíba: A – *Crassostrea *sp.; B – *Tagelus plebeius*; C – *Mytella guyanensis*; D – *Iphigenia brasiliensis*; E – *Pugilina morio*; F – *Anomalocardia brasiliana*. N.B. Molluscs D and E are occasionally captured (Photo: Guy Nishida).

The main molluscs exploited from mangrove ecosystems in Paraíba are: *Crassostrea rhizophorae *(Guilding, 1828) (Fig 3-A), attached to roots and stems of *Rhizophora mangle*, visible during low tides; *Crassostrea *sp., attached to pebbles, stones and other hard substrata in beds of rivers and river arms (known as 'gamboas'); *Mytella guianensis *(Lamark, 1819) (Fig 3-C), buried in the muddy surface of the mangrove habitat, forming banks; *Anomalocardia brasiliana *(Gmelin, 1791) (Fig. 3-F) and *Tagelus plebeius *(Ligthfoot, 1786) (Fig. 3-B), buried in the sandy-clay-loam banks of the estuary.

**Figure 4:**
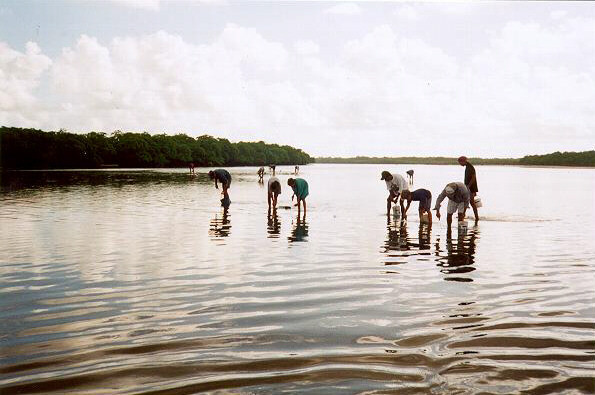
Gatherers using a wooden short-handled hood for capturing *Tagelus plebeius *during low tide, in the estuary of the River Paraíba do Norte (Photo: Guy Nishida).

The techniques the gatherers use for catching molluscs are carried out according to the species they catch. They use their bare hands and tools: a hoe, a straight blade sickle, and a wooden short-handled hook. They also catch molluscs by skin diving. In Fig. 4 we see several people gathering the mollusc *Tagelus plebeius*. Men, women, and children practise gathering activity, as was also reported elsewhere; Silva *et al*. [43] reported that in the estuary of the Formoso River, state of Pernambuco, 90% of gatherers are women and Costa Neto and Marques [21] reported that in the estuary of the Siribinha River, state of Bahia, the gatherers are men, women, and children.

**Figure 5:**
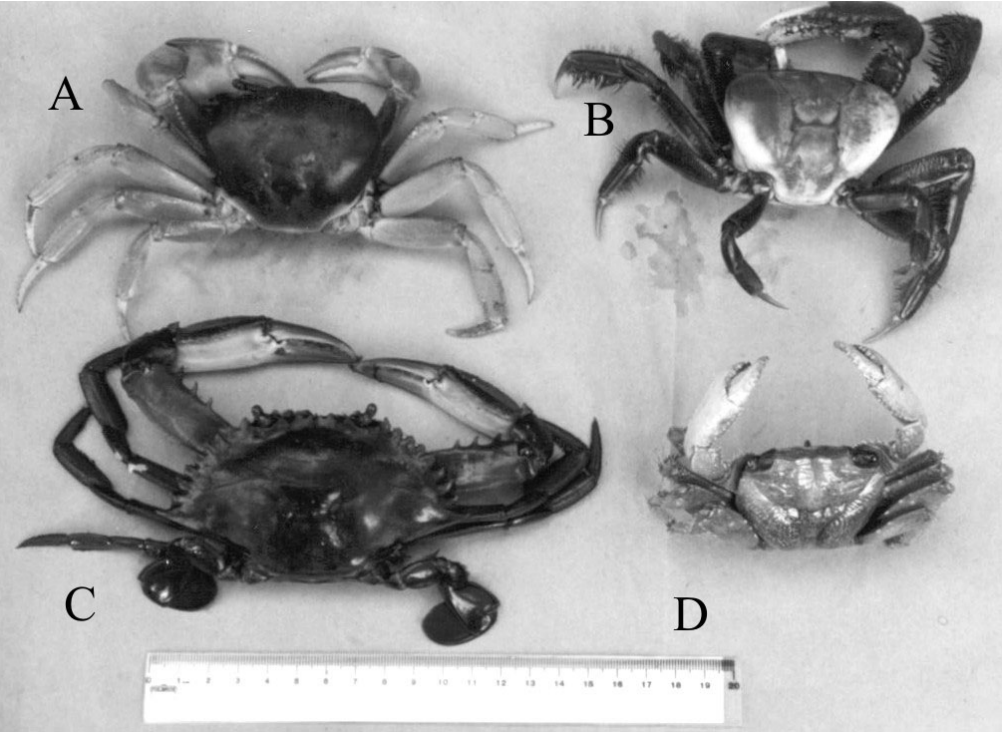
Crustacean exploited commercially in the littoral of the state of Paraíba: A – *Ucides cordatus*; B – *Cardisoma guanhumi*; C – *Callinectes danae *and D – *Goniopsis cruentata *(Photo: Guy Nishida).

The main species of crustaceans exploited are: *Cardisoma guanhumi *Latreille, 1825, *Goniopsis cruentata *(Latreille, 1803), *Callinectes *spp., and *Ucides cordatus *(L. 1763), (Fig. 5). The latter is the most relevant species to the economy of human communities on the coast of Paraíba [24,44].

Vergara Filho and Pereira Filho [45] reported men as predominant gatherers in the majority of the coastal states of Brazil, a result confirmed in the present work. Crustaceans are also collected by bare hand or by using tools especially adapted for catching them, or by using baits and traps. Canoes and rowing or sailboats, measuring between 5.0 and 7.0 m are commonly used by mollusc and crustacean gatherers. They enable the gatherers to navigate in shallow canals that cross the mangrove ecosystem.

**Figure 6:**
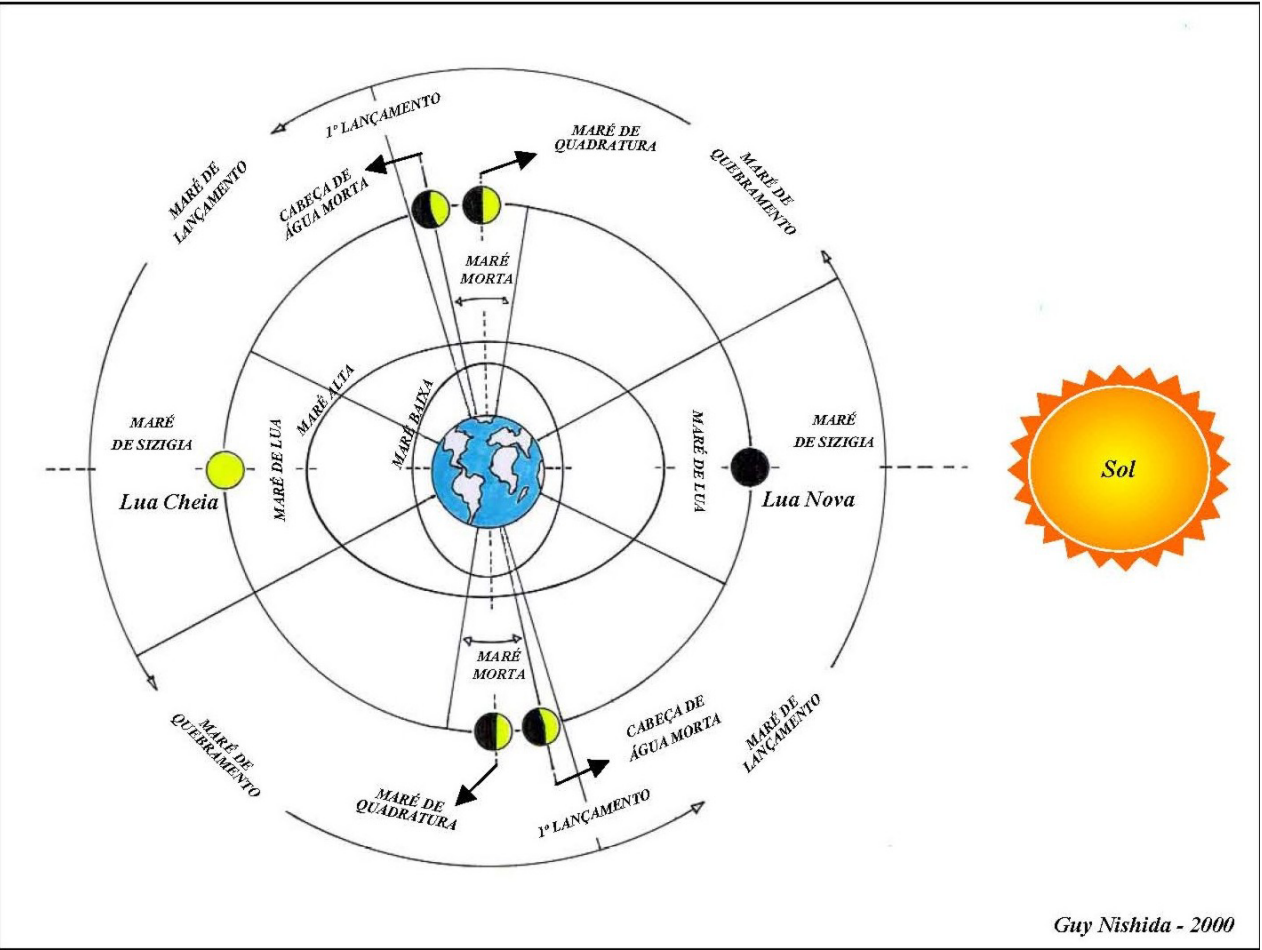
Diagram of the tide variations associated to the lunar cycle as viewed by crustacean and mollusc gatherers in the State of Paraíba.

The crustaceans and molluscs gatherers have a wide comprehension of the lunar cycle and its influence on tide variations, using it for organizing their collection activities. The gatherers' classification of tides associated with lunar cycle is shown in Fig. 6. The term used by gatherers for classifying the tides associated with lunar cycle are shown in Table 1. The knowledge on the phases of the moon is a critical factor for the survival of the traditional communities living on coastal zones exploiting the mangrove ecosystem resources, because the lunar cycle affects the biology of the animals and the gatherers collection activities [19].

**Table 1:** Terms used by crustacean and mollusc gatherers for classifying the tides associated with the lunar cycle.

*Term used	Meanings
'Maré de sizígia' or 'maré de lua'	'spring tide' (the greatest amplitude variations between high and low tide)
'Maré de quebramento'	'breaking tide' (the amplitude variation between the tides starts to decrease)
'Maré de quarto' or 'maré de quadratura'	'neap tide' (a small difference between high and low tides occurs, preceding the next situation)
'Maré morta'	'dead tide' (the minimum amplitude variation between the tides occurs in the days when the sun and the moon pull at right angles to the earth)
'Cabeça de água morta'	'head of dead water' (the last days of the 'dead tide' as the moon starts changing to waning crescent phase)
'Primeiro lançamento'	first 'flushing' (the amplitude variation between the tides starts to increase; it is the beginning of the 'flushing').

*Other terms used in Portuguese: sol = the sun; lua cheia = full moon; new moon = lua nova; maré alta = high tide; maré baixa = low tide.

Perkins [39] and Thurman [40] state that when the sun, the moon, and the earth are aligned (conjunction or opposition), the gravitational attraction forces are magnified, a situation the gatherers call 'maré de lua' (technically known in Portuguese as 'maré de sizígia') or spring tide, during which the highest amplitudes between high and low tides occur. For an approximate period of seven days the sun and the moon pull at right angles to the earth, causing a situation that is called by the gatherers as 'maré de quarto' (technically known in Portuguese as 'maré de quadratura') or neap tide. During neap tides, the smallest difference between high and low tides occurs because the gravitational pull is slight. For a few days near the neap tide, a minimum variation occurs between high and low tides, a situation the gatherers call 'maré morta', which literally means 'dead tide'; and at the end, when the moon starts changing to another phase (to waning crescent), the gatherers call 'cabeça de água morta', which literally means 'head of dead water' (Fig. 6).

In the littoral of Paraíba the tides are semi-diurnal, *i.e*. two high tides and two low tides at every 24 hours with maximum tidal level of 2.80 m. The variation between the tidal range as the moon changes from the waning crescent to new moon and from the waxing crescent to full moon, the gatherers call 'maré de lançamento', which literally means 'flushing tide' (Table 1). In this situation the water rises daily up to a maximum during the full or new moon. At this time the mangrove habitat is flooded quickly to a higher level during the high tide, and then is partly uncovered during the low tide, as observed by Nordi [10].

When the phases of the moon change from new moon to waxing crescent and from full moon to waning crescent, the gatherers say they have 'maré de quebramento', which literally means 'breaking tide', when the tides are reduced gradually at each day until minimum oscillations of tides happen (a situation similar to 'maré de quadratura' or neap tide) close to the quarters phases of the moon. At this time the extension of the mangrove habitat flooded during the high tide and uncovered during the low tide are smaller.

Costa Neto and Marques [21] reported that in the human community of Siribinha, in the state of Bahia, northeastern Brazil, the fishermen use similar terms to the ones recorded in the present study, *viz*. 'maré de lançamento', 'maré de quebramento', and 'maré morta', indicating changes of water currents. The authors reported that they anticipate the strength of a tidal flow by observing the time the moon rises and sets in the skyline. They also stated that according to duration and intensity of the moonlight available to nocturnal fishing, there are two lunar periods: 'maré de escuro', which literally means 'dark tide' and 'maré de lua', which literally means 'moon tide'. The fishermen say the former occurs from the waning crescent to the waxing crescent including the new moon, and the latter occurs from the waxing crescent to the waning crescent, including the full moon. Cordell [26] also reported similar terms among fishermen communities in southern Bahia.

The influence of the phases of the moon and the tide variations on activities of crab gatherers were reported by Alves and Nishida [19] and by Nordi [10]. Mollusc gatherers' attitudes were reported by Nishida [25]. According to Mourão [29] the tide movements are the main factors determining the fishing strategies in the estuary of the Mamanguape River. Santana [46] emphasizes that knowledge of environmental factors (moon phases, tidal movements, storm formation, types of wind and species behaviour) are very important to optimize fishing. She reported that fishermen in the Bahia decide on 'good or bad tides for fishing' according to the lunar conjunction.

Knowledge of phases of the moon and their influence on the movement and concentration of marine organisms is crucial to successful coastal fisheries [20]. Alves and Nishida [19] pointed out that gatherers of the land crab 'caranguejo uçá' (*Ucides cordatus*) in Paraíba associate the phases of the moon and the tide variations with the distinct phases of the life cycle of that crustacean, including ecdysis, mating, and spawning. These authors confirmed the gatherers' observation that the ecdysis of *U. cordatus *is influenced by the tidal cycle. Robertson [47] reported patterns of recruitment of some reef fishes in Panama, according to lunar cycles.

Costa Neto and Marques [21] emphasized the importance of the lunar cycle on the use of fishing equipment, reasoning that the cycle affects the behaviour of fishes and their human predators. Crab gatherers of *U. cordatus *for example, collect the animals during low tides, according to the phases of the moon [10]. Cordell [26] stated that the cyclic regularity of tides affects the mechanical operations of fishing methods and the distribution of species in the estuary. Thé [47] reported information from fishermen at Tres Marias dam, state of Minas Gerais, concerning changes in the reproductive behaviour and migration of fishes according to 'tidal forces'.

**Figure 7:**
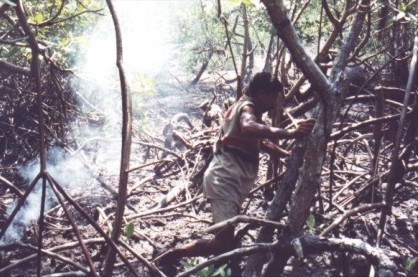
Gatherer of the land crab 'caranguejo uçá' (*Ucides cordatus*) when using the technique of 'tapamento' (fillup the burrow opening) (Photo: Rômulo Alves).

Crab gatherers use the most adequate technique according to prevailing tidal characteristics, whether 'maré de quebramento' ('breaking tide') or 'maré de lançamento' ('flushing tide'). During the former, gathering is performed in the mangrove ecosystem where *Rhizophora mangle *dominates. In this habitat and at this time, they use the 'braceamento' (the gatherer inserts his arm in the burrow and catches the crab by holding its dorsal carapace), which is quite efficient because the crab does not go deep in the burrow. If the crab goes deeper in the burrow, the collector uses the technique of 'tapamento' (Fig. 7), which literally means 'to fill up': as the crab enters the burrow the gatherer covers up the opening with mud and tree roots and after some time he returns to collect the crab. In this situation the crab will go to the sediment surface independently of the tides. However, the tidal conditions are still important for the gatherers because they would not distinguish burrows covered by water of high tides. Leite-Filho *et al.*[28] also reported the influence of the phases of the moon in the capture of lobsters.

Therefore, the knowledge of the lunar calendar by gatherers and fishermen is an adaptation by humans as they use appropriate techniques in the best moment for each species, as stated by Costa Neto and Marques [21]. These latter authors reported that on the littoral of the Bahia, according to perception and knowledge of local fishermen, nocturnal fishing by using casting nets are quite efficient at 'dark tides' (during the new moon). The use of 'waiting nets' in the estuary is preferable at 'moon tides' (during the full moon). Fishermen who use 'camboa' (some sort of enclosure or trap made of tree branches and foliage for capturing fishes) say that this device when applied during 'flushing tides' are less physically tiring than during 'breaking tides'. During the former they get up only once in the night to guard and clean the trap, while during the latter they guard and clean the trap at least three times in the night.

Fishers of Roviana Lagoon, Solomon Islands, organise their fishing activities according to a traditional lunar calendar they use to predict the movements, aggregations, and feeding behavior of target species. Some carangid species, for example, were said to concentrate in dense numbers within Roviana Lagoon around new and full moons. Fishers specifically target these fish during these lunar stages [49].

Molluscs collections are also performed during low tides, preferably during the 'maré de lua' or 'maré de sizígia' (spring tide) because the islets and mudbanks are not covered by water for as long a period as in the 'maré de quarto' or 'maré de quadratura' (neap tide). The frequency of the collections increases as neap tide progresses, and it decreases during the 'maré de quebramento' ('breaking tide'), reaching a minimum or coming to an end during the 'maré morta' ('dead tide').

Moss [50] reported that the Tlingit people, indigenous from northeast coast of Alaska, collect molluscs as food mainly in low tides during the new and full moon. Schaeffer-Novelli [51] reported that *Anomalocardia brasiliana*, a mollusc, was collected efficiently in large areas of the beach in the northern littoral of the state of São Paulo, southeastern Brazil, during the 'maré de sizígia' (spring tide).

The gatherers' perception of the lunar cycle and its influence on their strategies and techniques for capturing the animals in the estuary is reflected in their statement about mating and spawning of crustaceans and molluscs influenced by the tides, as confirmed in the works of Diele [52], Góes [53], Nishida [25] and Freire [54]. Molluscs gatherers in the estuary of the Paraíba do Norte River correlate the lunar cycle and the tides with the meat production (reflected by the condition index) of the mollusc *Tagelus plebeius*, as reported by Nishida [25].

The observations we performed showed that crustacean and mollusc gatherers of two important estuaries of Paraíba have an accurate knowledge of the phases of the moon and the consequent ebb and flow of the tides. The gatherers are acquainted with this cycle through their daily practice exploiting the natural resources, a knowledge that is transmitted verbally and perpetuated through generations.

As stated by Alves and Nishida [19] this wisdom may give important information to the proper management of natural resources, mainly of estuarine animals, whose preservation depends on an efficient legislation entirely compatible with the human communities exploiting those resources directly. Diegues *et al*. (1999) state that studies of ethnomanagement indicate that the link between traditional wisdom and scientific knowledge can achieve an effective preservation of Nature which will also be more fair.

In summary, the gatherers' knowledge on the influence of lunar-tide cycles on crabs and molluscs is extremely important in the littoral of Paraíba state where gatherers and fishermen organize their activities according to those cycles. Gatherers associate tidal variations to life cycle of different crustaceans and molluscs they exploit, e.g. they are able to recognize the influence of tides on female crabs' and molluscs' reproductive period. They also recognize that the distribution of those animal species in mangrove habitats and estuaries is directly related to variation of tides. Gatherers' knowledge can provide a useful basis for understanding local crab and mollusc stocks and their population dynamics. This kind of information may be used for establishing extractive reserves as well as for delimiting the 'defeso' (a period of time stipulated in Brazil during which a species can not be caught) and for establishing protected areas where animal species reproduce, aiming to maintain their natural stocks.

Ethnoecological studies may also help in promoting dialogue and cooperation between fishers and scientists. The literature illustrates the significance and value of traditional knowledge in Brazil. Alves and Nishida [19], for example, reported on the influence of tide variations on the ecdysis of the mangrove crab *Ucides cordatus *in natural environments, based on information obtained from gatherers.

Cultural values and perceptions arising from more experienced elder people should be taken into account before decisive action about their livelihoods be formulated by governmental authorities. Their contributions are essentially concerned with the sustainable management of the resource, which unfortunately have not yet been given satisfactory representation in the decision making process [55].
